# Lower vitamin D status is more common among Saudi adults with diabetes mellitus type 1 than in non-diabetics

**DOI:** 10.1186/1471-2458-14-153

**Published:** 2014-02-11

**Authors:** Nasser M Al-Daghri, Omar S Al-Attas, Majed S Alokail, Khalid M Alkharfy, Sobhy M Yakout, Naji J Aljohani, Hanan Al Fawaz, Abdulrahman SM Al-Ajlan, Eman S Sheshah, Mansour Al-Yousef, Mohammad Alharbi

**Affiliations:** 1 Biomarkers Research Program, Biochemistry Department, College of Science, King Saud University, Riyadh 11451, Saudi Arabia; 2 Prince Mutaib Chair for Biomarkers for Osteoporosis, Biochemistry Department, College of Science, King Saud University, PO box 2455, Riyadh 11451, Saudi Arabia; 3 Center of Excellence in Biotechnology Research Center, College of Science, King Saud University, Riyadh 11451, Saudi Arabia; 4 College of Pharmacy, King Saud University, Riyadh 11451, Saudi Arabia; 5 Faculty of Medicine, King Saud bin Abdulaziz University for Health Sciences, King Fahad Medical City, Riyadh 11525, Saudi Arabia; 6 College of Food, Science and Agriculture, King Saud University, Riyadh 11451, Saudi Arabia; 7 Riyadh College of Health Sciences, King Saud University, Ministry of Higher Education, Riyadh 11451, Saudi Arabia; 8 Diabetes Care Center, Prince Salman Bin Abdulaziz Hospital, Riyadh, Saudi Arabia; 9 Health Affairs for Riyadh Region, Ministry of Health, Riyadh, Saudi Arabia; 10 Diabetes Centers and Units Administration, Ministry of Health, Riyadh, Saudi Arabia

**Keywords:** Vitamin D, Vitamin D Deficiency, Type 1 diabetes

## Abstract

**Background:**

Vitamin D deficiency is an increasingly recognized comorbidity in patients with type 1 diabetes mellitus (DMT1), suggesting that vitamin D deficiency might play a role in DMT1. We aimed to determine and compare the vitamin D status of Saudi adults with and without DMT1.

**Methods:**

A total of 60 Saudi adults with DMT1 from the Diabetes Clinics and 60 non-DM, healthy controls were included in the study. The mean age for those with DMT1 was 25.9 ± 16.1 years versus 36.7 ± 3.6 years among the controls. We measured serum 25-hydroxy vitamin D (25OHD), calcium, cholesterol, blood glucose, HDL, and triglycerides and compared the results between the DMT1 group and control subjects.

**Results:**

Both the DMT1 and healthy groups had vitamin D deficiency. The mean levels of 25OHD were significantly lower in the DMT1 adults than in the controls (28.1 ± 1.4 nmol/L versus 33.4 ± 1.6 nmol/L). In the DMT1 adults, 66.7% were mildly, 31.7% moderately, and 3.3% severely vitamin D deficient as compared with 41.7% (mildly), 31.7% (moderately), and 5% (severely) in the control group. Overall, 100% of the DMT1 adults and 78% of the healthy children were vitamin D deficient.

**Conclusion:**

The prevalence of vitamin D deficiency among DMT1 adults was relatively high. Therefore, screening for vitamin D deficiency and supplementation for this population should be warranted.

## Background

Diabetes mellitus Type 1 (DMT1) is the most common life-threatening endocrine disorder in children and young adults worldwide and its incidence appears to be increasing. Originally coined juvenile diabetes or insulin-dependent diabetes, DMT1 traditionally has been considered a pediatric disease. In a recent multicenter US study, the incidence of DMT1 was approximately 20 per 100,000 among youth less than 20 years of ag with; no gender differences observed [1]. Currently, there are limited population-based estimates of DMT1 among adults; however, the medical literature suggests that up to 40% of new cases of DMT1 occur in persons over the age of 18 years [2,3]. The rise in the incidence of diabetes has broken the age limit boundaries and is currently seen in all age groups.

The observation of Adams and colleagues that symptomatic diabetes occurred more frequently at higher latitudes suggests that vitamin D deficiency might play a role in DMT1 [4,5]. Several reports showed a higher incidence of vitamin D deficiency in children and adolescents [6-9] with DMT1. However, studies examining vitamin D deficiency in DMT1 adults from the Middle East are limited. A recent study from Saudi Arabia revealed that the incidence of severe vitamin D deficiency was considerably higher in children with DMT1 [7,10]. The investigators have shown that the mean serum levels of vitamin D were significantly lower in children with DMT1 than non-diabetic children. However, both groups were in the mild to moderate vitamin D deficiency category [2]. Because of the high prevalence of vitamin D deficiency in children and adolescents with DMT1, some reports [4,6] suggested routine screening for vitamin D status in this population. Optimal vitamin D status and close follow-up were recommended [8,11].

Saudi nationals, particularly women, are not exposed to the sunlight long enough to synthesize vitamin D due to the culture requiring clothing to cover most of the body. Furthermore, many young people are not consuming sufficient fresh milk or dairy products fortified with vitamin D. Hence, it is important to investigate the vitamin D status and the risk of DMT1 in Saudis adults. The aim of this study is to assess the vitamin D status of in Saudi adults with DMT1 in comparison to healthy controls, and the relationship with glycemic control and duration of diabetes.

## Methods

### Subjects

The study sample included Saudi subjects recently diagnosed with DMT1 and those with established DMT1. Sixty subjects with DMT1 (more than 5 months duration), and 60 healthy control subjects were randomly and cross-sectionally selected. Written informed consent was taken from each subject before study inclusion. Ethics approval was granted by the Ethics Committee of the College of Science, King Saud University, Riyadh, Kingdom of Saudi Arabia (KSA). Participating subjects were recruited and enrolled in 4 primary health care centers (PHCCs) within the Riyadh Central Region. Study subjects were asked to complete a generalized questionnaire that contains demographic information including past and present medical history, and to return after fasting for more than 10 hours for anthropometry and blood withdrawal. At the screening visit, blood samples were examined for levels of sugar and cholesterol. Subjects who had abnormal levels of these at chemical laboratory tests were excluded.

### Anthropometry and blood collection

Subjects were requested to visit their respective PHCCs following an overnight fasted state (>10 hours) for anthropometry and blood withdrawal by the PHCC nurse and physician on duty, respectively. Anthropometry included height (rounded to the nearest 0.5 cm), weight (rounded to the nearest 0.1 kg), waist and hip circumference (centimeters), and mean systolic and diastolic blood pressure (mmHg, average of 2 readings). Body mass index (BMI) was calculated as weight in kilograms divided by height in square meters. Fasting blood samples were collected and transferred immediately to a non-heparinized tube for centrifugation. Collected serum was then transferred to pre-labeled plain tubes, stored on ice, and delivered to the Biomarkers Research Program (BRP) in King Saud University, Riyadh, KSA, for immediate storage at −20°C.

### Sample analyses

Fasting glucose, lipid profile, calcium, and phosphorous were measured using a chemical analyzer (Konelab, Espoo, Finland). Serum 25(OH)D was measured with a Roche Elecsys modular analytics Cobas e411 using an electrochemiluminescence immuno assay (Roche Diagnostics, GmbH, Mannheim, Germany) and commercially available IDS kits (IDS Ltd, Boldon Colliery, Tyne & Wear, UK). The inter- and intra-assay coefficients of variation (CV) for 25 (OH) D ELISA were 5.3% and 4.6%, respectively, with 100% cross-reactivity to 25 (OH) D3 and 75% cross-reactivity to 25 (OH) D2. It should be noted that the BRP laboratory is a participating entity in the Vitamin D External Quality Assessment Scheme (DEQAS), and Quality Assurance (QA) standards are maintained by ISO 9000 and 17025, whereas the QA department audits the BRP laboratory at regular intervals.

### Data analysis

Data were analyzed using the Statistical Package for the Social Sciences version 16.0 (SPSS, Chicago, IL, USA). Normal continuous variables were presented as mean ± standard deviation. The paired *t*-test was used to compare differences between baseline and 1 year. Spearman’s correlation was done using osteocalcin and serum CrossLaps as dependent variables. Significance was set at *p* < 0.05.

## Results

We assessed clinical and laboratory findings in 60 adults with DMT1. The characteristics of the whole group according to DM status and vitamin D levels are shown in Table 1. Anthropometric parameters including age, gender, height, and weight were obtained (Table 1). Of the total number of subjects, 56.6% of the DMT1 subjects and 53.3% of the healthy adults were males, and 43.4% of the DMT1 subjects and 46.7% of the healthy adults were females. The mean age for the DMT1 subjects was 25.9 ± 16.1 years versus 36.7 ± 3.6 years for the healthy controls. Both groups differed significantly in all anthropometric measures. Adults with DMT1 had significantly lower mean BMI and waist and hip circumference (*p* < 0.001), but higher waist to hip ratio (WHR) than the healthy adults. DMT1 adults also had significantly higher fasting glucose concentrations and HDL-cholesterol (*p* < 0.001) than healthy adults. Calcium levels showed statistically significant differences between DMT1 and non-diabetic subjects (*p* = 0.01), though all subjects had calcium levels within the normal range. Calcium levels were 2.4 ± 0.36 mmol/L in the healthy controls and 2.2 ± 0.035 mmol/L in the DMT1 adults. Lips classified a level of 25-OH vitamin D above 50 nmol/L (20 ng/mL) as normal, 26–50 nmol/L (10–20 ng/mL) as a mild deficiency, 12.5–25 nmol/L (5–10 ng/mL) as moderate, and <12.5 nmol/L (<5 ng/mL) as a severe deficiency [12]. According to this classification, both groups had vitamin D deficiency and vitamin D levels were significantly lower among T1DM cases compared with healthy adults (*p* = 0.03). Mean 25OHD levels were 33.4 ± 1.6 nmol/L in the normal controls and 28.1 ± 1.4 nmol/L in the T1DM group (*p* = 0.03). Overall, 100% of the DMT1 and 78% of the healthy adults were vitamin D deficient.

**Table 1:** Basic characteristics and vitamin D levels in diabetic and healthy control adults

	**Control**	**Type 1 diabetes**	***P *** **value**
***N***	**60**	**60**	
Gender (M/F)	32/28	34/26	0.71
Age	36.7 ± 3.6	25.9 ± 16.1	<0.001
BMI (kg/m^2^)	28.2 ± 6.8	26.4 ± 6.1	0.12
Waist (cm)	83.1 ± 13.2	71.4 ± 14.1	<0.001
Hips (cm)	96.1 ± 16.5	80.1 ± 27.0	0.001
Waist-hips ratio	0.96 ± 0.16	0.87 ± 0.18	0.004
Cholesterol (mmol/L)	4.9 ± 1.0	5.0 ± 1.1	0.78
Glucose (mmol/L)	5.2 ± 0.63	13.8 ± 7.7	<0.001
HDL (mmol/L)	0.84 ± 0.34	1.1 ± 0.42	0.002
Triglyceride (mmol/L)	1.5 ± 0.74	1.4 ± 0.86	0.97
LDL (mmol/L)	3.4 ± 0.95	3.5 ± 0.91	0.81
Vitamin D (nmol/L)	33.4 ± 1.6	28.1 ± 1.4	0.03
Vitamin D deficiency (%)			
50-25 nmol/L	25 (41.7)	40 (66.7)	0.19
25.0-12.5 nmol/L	19 (31.7)	19 (31.7)	
<12.5 nmol/L	3 (5)	2 (3.3)	
Calcium (mmol/L)	2.4 ± 0.36	2.2 ± 0.35	0.01
Fish (g/week)	724.9 ± 163.7	150 ± 52.9	<0.001
Liver	224.5 ± 141.2	360 ± 203.2	0.13
Milk			
0 glasses/day	26 (43.4)	32 (53.3)	0.16
1-2 glasses/day	25 (41.6)	25 (41.7)	
3-4 glasses/day	9 (15.0)	3 (5.0)	

Daily consumption of vitamin D-rich foods was focused on milk drinking and fish consumption. DMT1 patients consumed significantly lower amounts of fish than healthy patients. The average daily intake of 3–4 glasses/day of milk was less in the DMT1 subjects than the healthy adults. As shown in Table 2, vitamin D levels were not associated with any parameters, but a weak negative association was found with triglycerides (R = −0.18, *p* = 0.04).

**Table 2:** Partial correlations with vitamin D as the dependent variable controlling for age

	**Over all**		**Control**		**T1DM**	
	**R**	***P *** **value**	**R**	***P *** **value**	**R**	***P *** **value**
**N**	**137**		**60**		**77**	
Waist (cm)	−0.07	0.39	0.01	0.94	−0.06	0.57
Hips (cm)	−0.11	0.22	−0.03	0.83	−0.07	0.53
Total cholesterol (mmol/L)	0.03	0.75	−0.03	0.82	0.05	0.68
Glucose (mmol/L)	0.12	0.16	0.09	0.50	0.03	0.78
HDL-cholesterol (mmol/L)	0.03	0.72	−0.05	0.72	0.08	0.48
Triglycerides (mmol/L)	0.18	0.04	0.27	0.04	0.02	0.85
LDL-cholesterol (mmol/L)	−0.15	0.16	−0.12	0.36	−0.19	0.29
Ca (mmol/L)	−0.07	0.41	0.01	0.97	0.14	0.24

*p*-value <0.05 is significant.

## Discussion

To the best of our knowledge, there are no population-based studies that have examined the association between vitamin D and DMT1 in young Saudi adults. Our participants with DMT1 all had vitamin D deficiency. The study revealed that vitamin D deficiency was considerably higher in T1DM adults (100%) compared with non-diabetic patients (78%). A significant difference in the mean value of vitamin D between the T1DM and healthy adults was found (*p* = 0.03). Although vitamin D deficiency was prevalent in both groups, it was much higher among the diabetic adults. We believe that vitamin D, in some fashion, might inhibit the autoimmune reaction targeted toward cells of the pancreas [13]. Furthermore, impairment of immune system function by a sub-optimum vitamin D status in infancy could have long-term effects on immune responses later in life.

These results support other studies demonstrating that vitamin D deficiency increases the risk of T1DM [14]. The prevalence of vitamin D deficiency was higher in our cohort (100%) than in previous Western studies [8,15] analyzing subjects with T1DM. The prevalence of vitamin D deficiency was 60.5% in a Swiss study [11], 43% in an Australian study [15], approximately 25% in an Italian study [16], and 15% in a North American study [8]. In Qatari children, a study revealed that vitamin D deficiency was considerably higher in children with T1DM (90.6%) compared with non-diabetic children (85.3%) [17]. In Australian children and adolescents with T1DM, the mean 25OHD was 64.6 nmol/L (61.3–67.9) in normal children and 54.7 nmol/L (50.3–58.9) in children with T1DM [15]. The proportions of 25OHD deficiency were 18% in normal children and 43% in children with T1DM [15]. In a Swedish study, the mean 25OHD levels were 96.7 ± 2.7 nmol/L for the control group and 82.5 ± 1.3 nmol/L for those with T1DM [18]. In a North American study, 25OHD levels were measured in 128 youths with T1DM. Fifteen percent were vitamin D deficient and 61% were vitamin D insufficient. Adjusted 25OHD mean levels were 26.8 + 6.7 ng/dL. The investigators showed that vitamin D deficiency was more prevalent among older children and those with a longer duration of diabetes [8]. In a Swiss prospective cross-sectional study, serum 25OHD levels were measured in 129 diabetic children [11]. Sixty percent were vitamin D deficient with a mean 25OHD level of 28.8 nmol/L (26–31.6); the number rose to 84% during winter, whereas there was no correlation with diabetes control [11]. These variations might be explained by differences in dietary intake, sun avoidance behaviors, geographical environment, skin color, or genetic predisposition.

Not all studies showed lower vitamin D levels in persons with T1DM compared with the control group. Serum samples from 110 subjects with T1DM and 153 control subjects were cross-sectionally analyzed and the 25OHD levels were similar among the 2 groups [19]. The median 25OHD level was 20.1 ng/mL (13–37.4) in the control group and 23 ng/mL (13.8-33.9) in the T1DM group. However, both groups had sub-optimal 25OHD levels [19]. Littorin et al., [18] found low 25OHD levels in 459 Swedish patients aged between 15 and 34 years who were newly diagnosed with T1DM compared with age- and location-matched controls. Another study from Italy [16] found low 25OHD levels in 88 newly diagnosed children and adolescents. A recently published paper from India reported similar results [20]. Understanding the nature of low vitamin D levels in adults with T1DM is important because it may potentially clarify the mechanisms of autoimmune ß-cell destruction, leading to interventions for the prevention or delay of insulin dependence by using vitamin D or its analogues.

Several limitations were faced while this current study was conducted, including the exact duration and time of sun exposure, which might affect the results. In our study, mean 25OHD levels in diabetic adults were relatively lower than those published in Western studies [8,11,15]. This might reflect the high prevalence of 25OHD deficiency and insufficiency in our normal population. The high prevalence of vitamin D deficiency might be related to decreased sun exposure. Despite the fact that there is ample sunlight throughout the year, in the Middle East and other Arab countries, time spent outdoors is severely limited. Thus, vitamin D deficiency was highly prevalent in Saudi population. It has been documented in a few studies [21,22] that vitamin D status showed a strong relationship with lifestyle, especially type of clothing worn. Vitamin D status was much better in women with Western clothing than in women with traditional veils with the face and hands covered. Vitamin D deficiency was particularly prevalent among veiled women in Turkey, Lebanon, Jordan, Saudi Arabia, and Iran [21]. The Saudi population covers their body completely except their faces. Wearing concealing clothing and restriction of outdoor activities has been reported previously as a risk factor for vitamin D deficiency in Saudi Arabian adolescents [23].

## Conclusion

The present study revealed that vitamin D deficiency was higher in adults with T1DM compared with non-diabetic controls. Moreover, vitamin D deficiency was found to be common among the Saudi population. The low levels of vitamin D in the young population have been attributed mainly to social customs, particularly the avoidance of sunlight and vitamin D supplements. It will be of interest for future studies to investigate whether vitamin D supplementation improves glycemic control in vitamin D deficient diabetic adults. Additionally, we suggest the need for a randomized controlled trial to assess the correlation between vitamin D with control and prevention of T1DM.

## Competing interests

The authors declare that they have no competing interests.

## Authors’ contributions

NMA, OSA, MSA and KMA conceived the study. SMY, EAS, MA and HA carried out data acquisition and interpretation. SMY, ASM and NJA analyzed the data and prepared the manuscript. SMY, MA and NMA drafted and revised the final version of the manuscript. All authors provided intellectual contributions to the manuscript and has read and approved the final version.

## Pre-publication history

The pre-publication history for this paper can be accessed here:

http://www.biomedcentral.com/1471-2458/14/153/prepub
